# Giant phytobezoar; an unusual cause of gastric outlet obstruction: A case report with literature review

**DOI:** 10.1016/j.ijscr.2020.02.012

**Published:** 2020-02-07

**Authors:** Sahir Mahir, Abdulwahid M. Salih, Okba F. Ahmed, Fahmi H. Kakamad, Rawezh Q. Salih, Shvan H. Mohammed, Drood C. Usf, Hemn A. Hassan, Fakher Abdulla

**Affiliations:** a Department of Surgery, Al-Jamhori Teaching Hospital, Mosul, Iraq; b University of Sulaimani, College of Medicine, Department of Surgery, Sulaimani, Kurdistan Region, Iraq; c Mosul Cardiac Center, Mousl, Iraq; d Kscien Organization, Hamdi Str. Azadi Mall, Sulaimani, Kurdistan Region, Iraq; e Shar Medical Center, Laboratory Department, Ibrahem Pasha Street, Sulaimani, Kurdistan, Iraq; f Chara Laboratory, Shahedan Street, Kalar, Kurdistan, Iraq

**Keywords:** Bezoar, Gastric outlet obstruction, GOO, Phytobezoar, Gastrotomy, Giant

## Abstract

**Introduction:**

Phytobezoar which is described as an undigested or incompletely digested food. It is an odd cause of gastric outlet obstruction (GOO). The aim of this study is to present and discuss a case of GOO caused by multiple giant bezoars.

**Case report:**

A 24-year-old female, presented with abdominal pain and vomiting (non-bilious) with negative past history. Examination and investigations revealed multiple giant bezoars requiring emergent surgical intervention. An exploratory laparotomy was conducted. Two giant bezoars were palpated in the stomach and removed through an anterior gastrotomy.

**Discussion:**

Bezoars are regarded as rare benign causes of GOO. Bezoars can present with vomiting, nausea, and/or symptoms of GOO. Predisposing risk factors include delayed gastric emptying (as in case of diabetic mellitus) vagotomy, partial gastrectomy pyloroplasty, peptic ulcer disease, chronic gastritis, Crohn’s disease, and carcinoma of the gastrointestinal tract. The current case had phytobezoar without any known risk factor.

**Conclusion:**

GOO caused by phytobezoar can occur in patients without history of previous gastric surgery or diabetes mellitus. Urgent laparotomy may be indicated.

## Highlights

• Phytobezoar is described as an impacted indigested or incompletely digested vegetable and fruit fibers.
• Bezoar is a rare cause of gastric outlet obstruction. Being multiple and giant is even rarer.
• The aim of this study is to report a case of unusual gastric outlet obstruction by two giant bezoars.

## 1 Introduction

Phytobezoar causing GOO is a rare disease. Phytobezoar is described as an impacted indigested or incompletely digested vegetable and fruit fibers. Phytobezoar is an infrequent late complication of a previous gastrointestinal operation [1]. Hypoacidity and gastric motility disorders after operation of the stomach are the basis of bezoar formation. These result in compromised gastric emptying even gastroparesis and/or dwindling acid production [2]. Depending on the location and size, signs and symptoms of gastrointestinal bezoars vary, such as abdominal distension, abdominal pain, anemia—or even upper gastrointestinal bleeding—or signs and symptoms of intestinal obstruction because of large intestinal bezoars [3]. Bezoar is a rare cause of GOO. Being multiple and giant is even rarer [4]. The aim of this study is to report a case of unusual GOO by two giant bezoars in line with SCARE guideline with a brief literature review [5].

### 1.1 Patient information

A 24-year-old female brought to the emergency department with abdominal pain and vomiting (non-bilious) for three day duration. The character of the pain was colicky in nature associated with nausea. She had no oral intake for two days because of pain. She reported history of mild dyspepsia, weight loss, and early satiety for which she used to take irregular anti-acid medications few months before presentation. The condition started to deteriorate in the last 3 days. There was negative past medical and past surgical history.

### 1.2 Clinical findings

The patient was fully conscious, mildly dehydrated and have neither pallor nor jaundice. Heart rate was 96 beats/minute, regular with good volume, blood pressure was 100/70 mmHg and respiratory rate was 22 cycles/minutes. She had an evident uneven abdominal swelling with a centrally upturned umbilicus. Abdomen was soft on palpation, with a left hypochondrial intra-abdominal, firm, elliptical, smooth surface, mobile, not tender, not compressible, not pulsatile mass, extending to the epigastric region, measuring about 20 cm × 10 cm. Bowel sound was normal. Both rectum (by digital examination) and hernia orifices were empty.

### 1.3 Diagnostic assessment

**Fig. 1:**
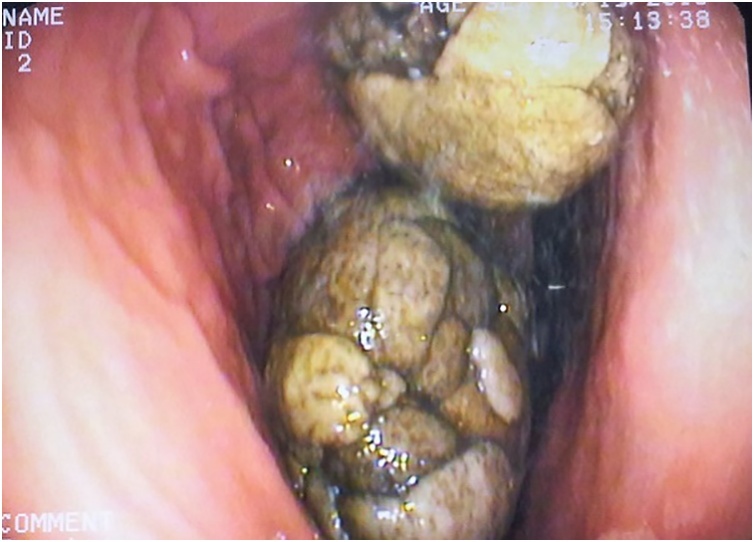
Endoscopic findings of the bezoar.

Laboratory findings demonstrated the followings: Hemoglobin:13 g/dl, packed cell volume: 36%; white blood cells: 9 × 10^9^ cells/L; erythrocyte sedimentation rate: 14 mm/hour; blood urea: 6.8 mmol/L; serum creatine: 113 μmol/L; serum potassium: 4.2 mmol/L; serum sodium: 138 mmol/L. Abdominal ultrasound displayed a large upper abdominal mass. Esophagogastroduodenoscopy (EGD) discovered two giant, tough, bezoars. The largest one extended from the gastric fundus to the pylorus, and took the shape of the stomach. A smaller one was round and gray. There was no evidence of gastric ulcer or gastritis. The endoscopy could not scope the duodenum. (Fig. 1). The bezoars were hard in consistency which were failed to be dug out by the endoscopy.

### 1.4 Therapeutic intervention

**Fig. 2:**
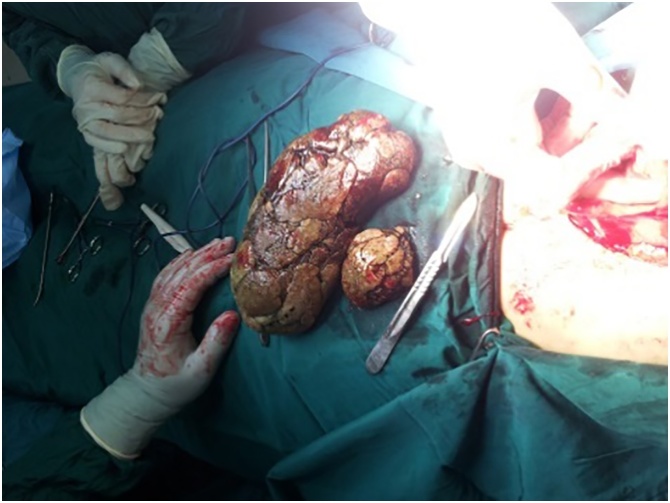
Intraoperative finding of the specimen.

Under general anesthesia, in supine position, the patient underwent emergency laparotomy through an upper midline incision. There were two intra-gastric masses. The bezoars were pulled out through a longitudinal gastrostomy (Fig. 2). The gastrotomy was closed in two layers. Oral feeding was commenced in the second postoperative day and the patient was discharged in the fifth postoperative day uneventfully.

### 1.5 Follow-up and outcomes

Postoperatively, she was put on oral analgesic and antibiotics for one week. She was followed up for eight months, the wound was healthy.

## 2 Discussion

GOO is a clinical syndrome implies to any disease that mechanically impedes gastric emptying. The causes may be benign or malignant diseases. In the previous era, peptic ulcer was the most frequent cause of GOO, accounting for up to 90% of cases. However, the incidence has declined with the discovery of the Helicobacter Pylori and proton pump inhibitors. Currently, 50–80% of cases have been attributable to malignancy [6]. Samad and associates published their 6-year experience with GOO. Among 52 patients, they found malignancy in 35% of the cases [6]. The benign etiology includes gastric polyps, gall stone, pyloric stenosis, congenital duodenal webs and pancreatic pseudocysts [7]. Bezoars are regarded as rare benign causes of GOO [7]. They are categorized into 4 groups. These include intensive plant fibers (phytobezoar), milk curds (lactobezoar), swallowed hair (trichobezoar) and medications (pharmacobezoar) [8,9]. Predisposing risk factors include delayed gastric emptying (as in case of diabetic mellitus) vagotomy, partial gastrectomy pyloroplasty, peptic ulcer disease, chronic gastritis, Crohn’s disease, and carcinoma of the gastrointestinal tract [4]. The current case had phytobezoar without any known risk factors.

Bezoars can present with vomiting, nausea, and/or symptoms of GOO [10]. However, symptoms such as upper gastrointestinal bleeding, intestinal obstruction and GOO are rather infrequent [11]. This patient had features of GOO with the symptom of emesis immediately after intake of non-liquid food and even with liquid food later in the course of the disease. The obstruction was confirmed by endoscopy and the surgical procedure. They composed of grey, hard, food fibers. We excluded persimmon phytobezoar because it is not commonly used by our population.

The treatment options include (1) conservative, in which the patients are prescribed prokinetic and enzymatic dissolvents like cellulose, papain, acetylcysteine and Coco-Cola or mechanical disruption such as endoscopic fragmentation, gastric lavage or extracorporeal lithotripsy. Small bezoar could be treated conservatively [12,13]. Endoscopic fragmentation is needed if the size of bezoar ≧ 3 cm, followed by extraction of those fragments which are larger than one centimeter to prevent the risk of intestinal obstruction. Even large gastric bezoars, when uncomplicated, endoscopic removal can be applied [12,14]. Ugenti et al. reported a 10-cm bezoar in a 76 year old male causing pressure ulcer, they succeeded in the fragmentation of the foreign body [15]. On the other hand, Mohammed and colleagues did not support endoscopic procedure for retrieval of bezoars especially large size as it needs frequent trials leading to mucosal erosion with subsequent esophagitis and gastritis [9]. It has been reported that a case of intestinal obstruction was resulted from a phytobezoar after chemical dissolution for a large phytobezoar in the stomach [16]. (2) Operative extraction is usually selected as the initial therapy for patients with GOO, because gastric bezoars presenting with GOO are generally too large to be retrieved and too hard to be broken with a rapid development of electrolyte imbalance [17,18]. Although it is evident from most of the previous reports that nonsurgical treatment could be safe and effective for bezoars, operation—which can rule out the advancement of serious complications—should be considered for multiple giant bezoars with gastric outlet obstruction like this case like the current case [19].

The recurrence rate of gastric bezoars has been reported to be around 14% [19]. To prevent reformation of the bezoar, these patients should minimize or avoid intake of particular fibers in food especially citrus, persimmon fruits and vegetable fibers. They should grind bolus perfectly, use prokinetics medications like metoclopramide for patients with gastrointestinal dysmotility and behavioral therapy plus selective serotonin receptor inhibitor or tricyclic antidepressants treatment for trichotillomania vegetable fibers [20].

## 3 Conclusions

Phytobezoar can happen in patients without history of previous gastric surgery or diabetes mellitus, and should not be under estimated. Early diagnosis and treatment is very important to save the patient life and prevent recurrence. Although pharmacotherapy (chemical resolution) and endoscopy are the excellent treatment options, surgery (laparoscopy and laparotomy) still play an important role in certain circumstances.

## Sources of funding

No source to be stated.

## Ethical approval

Approval is not necessary for case report in out locality.

## Consent

Consent has been taken from the patient and the family of the patient.

## Author contribution

Sahir Mahir: Surgeon performed the operation and follow up.

Okba F. Ahmed, Shvan H. Mohammed, Fahmi H. Kakamad, and Rawezh Q. Salih: Writing the manuscript and follow up.

Abdulwahid M. Salih, Drood C. Usf, Hemn A. Hassan and Fakher Abdulla: literature review, final approval of the manuscript.

## Registration of research studies

Not applicable.

## Guarantor

Fahmi Hussein kakamad.

## Provenance and peer review

Not commissioned, externally peer-reviewed.

## Declaration of Competing Interest

There is no conflict to be declared.
